# Analysis of Geometrical Characteristics and Properties of Laser Cladding 85 wt.% Ti + 15 wt.% TiBCN Powder on 7075 Aluminum Alloy Substrate

**DOI:** 10.3390/ma11091551

**Published:** 2018-08-28

**Authors:** Yu-Xin Li, Peng-Fei Zhang, Pei-Kang Bai, Zhan-Yong Zhao, Bin Liu

**Affiliations:** School of Materials Science and Engineering, North University of China, Taiyuan 030051, China; zhangpf1007@163.com (P.-F.Z.); baipeikang@nuc.edu.cn (P.-K.B.); syuzzy@126.com (Z.-Y.Z.); liubin3y@nuc.edu.cn (B.L.)

**Keywords:** aluminum alloy, laser cladding, geometrical characteristics, microstructure, properties

## Abstract

Ti/TiBCN composite coatings were prepared on a 7075 aluminum alloy surface by laser cladding. The relation between the main processing parameters (i.e., laser power, scanning speed, and powder feeding rate) and the geometrical characteristics (i.e., height, width, penetration depth, dilution and wetting angle) of single clad tracks is studied by linear regression analysis. The microstructure, micro-hardness and electrochemical corrosion were investigated by scanning electron microscopy, a Vickers micro-hardness machine, and a standard three-electrode cell, respectively. The results showed that all geometrical track characteristics are observed with high values of the correlation coefficient (R > 0.95). In addition, the average hardness value (750 HV_0.2_) was obtained of the Ti/TiBCN composite coating, and polarization curves indicated that the composite coatings were harder to corrode than the substrate.

## 1. Introduction

Laser cladding is an advanced surface modification technique, which provides thick protective coatings with a high quality on substrates. It is always used to modify the surface properties of various metal materials [1]. Compared to conventional surface treatment techniques (plasma spraying, sol-gel method [2], chemical deposition, physical vapor deposition [3], electroplating and thermal spraying [4]), the laser cladding technique results in good metallurgical bonding, formation of a dense microstructure and lower metallurgic defects [5,6].

The operational window for the laser cladding process is usually interpreted in terms of processing parameters, i.e., laser power (P), energy distribution, scanning speed (S), laser spot size, powder feeding rate (F), powder particle size, and the sort of shielding gas, etc. [7,8]. P, S and F have a significant effect on the clad geometry and properties of the coating [9]. A comprehensive description of the laser cladding process is quite complicated. Therefore, it is still necessary to study the relation between the main processing parameters (P, S and F) and the geometrical characteristics. Furthermore, many researchers have done this work. Ocelík et al. [10] studied the correlations between P, S and F and the geometrical characteristics of laser cladding Co powder on cast iron substrates. Riveiro et al. [11] studied the geometrical characteristics of laser cladding Al on 304 stainless steel substrates using linear regression analysis. Cheikh et al. [12] studied the effect of P, S and F on the cross-sectional characteristics. Barekat et al. [8] studied the geometrical characteristics of Co-Cr-Mo powder coating on a γ-TiAl substrate by linear regression analysis. Ansari et al. [13] studied the geometrical characteristics of NiCrAlY powder coating on Inconel 738 superalloy by laser cladding. Rashid et al. [14] analyzed the effect of the geometrical features on clad width, clad thickness, and depth of penetration into the substrate, and reported the dimensions of the heat-affected zone (HAZ). Nazari et al. [15] studied Ti-Fe coating on a titanium substrate. The result showed that the coating was composed of TiO_2_, TiC, Fe_2_O_3_, and Fe_2_C phases. The hardness of the coating (~800 HV) is two times higher than that of titanium substrate (~380 HV).

Aluminum alloy is one of the most important engineering materials and used in aerospace industries, particularly for automobiles, steamships and other fields, due to its low density, good castability, high thermal conductivity and machinability [16,17]. However, due to low surface hardness and high wear, the use of aluminum alloy is limited. To overcome such problems, laser cladding is an efficient approach to improve the performance of aluminum alloy [18]. At present, the laser cladding technique of aluminum alloy has been widely investigated by a number of investigators. In some research, ceramic materials are used as wear-resistant coating, such as SiC, B_4_C, Al_2_O_3_ and TiC particles [19,20,21]. TiBCN is a new ceramic material with high hardness, good corrosion resistance, good abrasion resistance, and good chemical stability [22,23,24,25]. However, there is very little research on Ti/TiBCN laser cladding coatings, especially into the influence of processing parameters on the geometrical characteristics of Ti/TiBCN coatings on 7075 aluminum alloy.

In the present paper, firstly, we explore the relations between P, S and F and the geometrical parameters of Ti/TiBCN composite coatings by linear regression analysis so as to propose an empirical guideline for Ti/TiBCN powder on 7075 aluminum alloy by laser cladding. Afterwards, perfect Ti/TiBCN composite coatings are fabricated, and their hardness and corrosion resistance were investigated in order to improve the properties of the 7075 aluminum alloy surface.

## 2. Materials and Methods

In the study, the materials used were 7075 aluminum alloy (30 mm × 15 mm × 10 mm), Ti powder (100–150 μm) and TiBCN powder (100–150 μm) for the substrate and the cladding coating, respectively. The mass of Ti/TiBCN powder was 85 wt.% and 15 wt.%, respectively. The chemical composition of 7075 aluminum alloy is given in Table 1.

The specimens for laser cladding were processed on a wave semiconductor laser (LDF4000-100, Laserline Gmbh, Mülheim-Kärlich, Germany) with a spot diameter of 1.5 mm, focal length of 150 mm, and the wavelength of 980–1020 nm. The robot ABB (ABB Engineering Ltd., Shanghai, China) was connected to the laser cladding system. The powder was delivered coaxially by a coaxial nozzle DMS-3D (Duomu Industry Co., Ltd., Shanghai, China). The geometry of the nozzle was a taper type with four channels. High-purity argon gas was used as a protective gas during the cladding, and the detailed parameters are presented in Table 2.

After laser cladding treatment, the specimens were sectioned in the transverse direction. Transverse sections were polished with SiC paper, and etched with a solution of 2 mL HF, 3 mL HCl, 5 mL HNO_3_ and 190 mL water. The clad geometry was characterized using scanning electron microscopy (SEM, INSPECTF50, FEI, Hillsboro, AL, USA). The micro-hardness was measured by a HVS-1000 Vickers hardness tester with load of 0.98 N and loading time of 15 s. The CHI660E electrochemical workstation was used to test the corrosion resistance of the coating in 3.5% NaCl solution.

**Figure 1:**
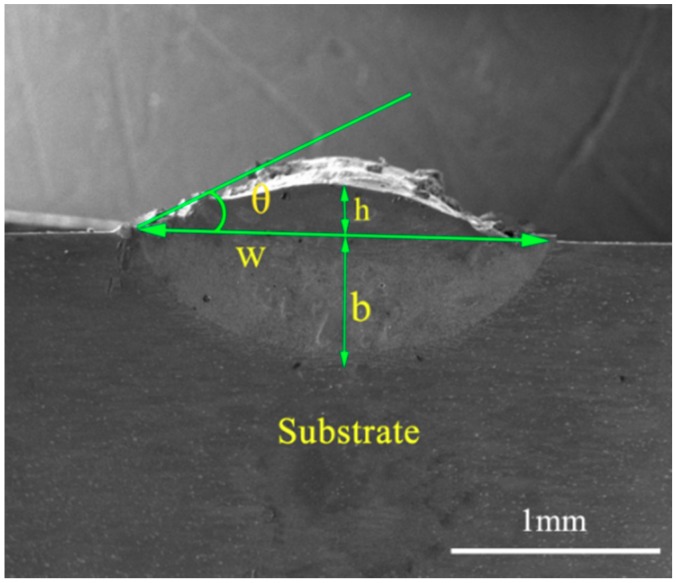
A schematic view of typical laser track with its main geometric features.

The shape of the tracks was measured by the MIAPS (Release version 5.7, Precise Instrument Co., Ltd., Beijing, China) software from transverse sections digital images. The measurements included the clad height (h), clad width (w), clad depth (b) and the angle of wetting (θ). A schematic view of typical laser track with its main geometric features is given in Figure 1. The dilution (D) is calculated by Equation (1) [26]:

(1) $$D(\%)=\frac{b}{b+h\;}$$

The different geometrical characteristics were predicted by the multiple regression analysis method. The mathematical model was established by Equation (2):

(2) $$y=A(P^{\alpha }S^{\beta }F^{\gamma })+B\;$$

where y represents the geometrical characteristics of measured values, *α*, *β*, *γ* are used to determine the linear regression analysis, A, B represent a constant. Take the logarithm of both sides, and get Equation (3):

(3) Iny=InA+αInP+βInS+γInF+InB 

If y_1_ = Iny, X = InP, Y = InS, Z = InF, C = InA + InB, as follows in Equation (4):

(4) $$y_{1}=\alpha X+\beta Y+\gamma Z+C\;$$

The value of *α*, *β*, *γ* and correlation coefficient R^2^ were calculated using SPSS Statistics 19 statistical analysis software (Version 19, IBM corporation, New York, NY, USA).

## 3. Results and Discussion

### 3.1. Geometrical Characteristics and Microstructure

**Figure 2:**
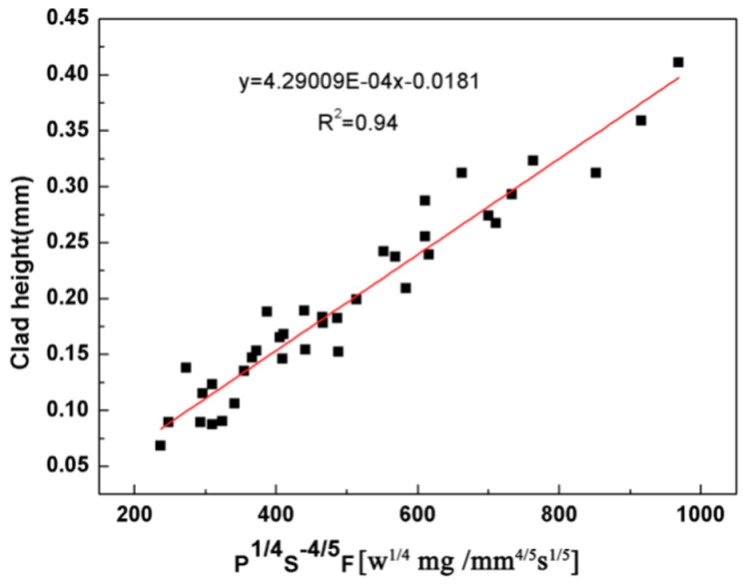
Relationship between the clad height and P^1/4^S^−4/5^F.

**Figure 3:**
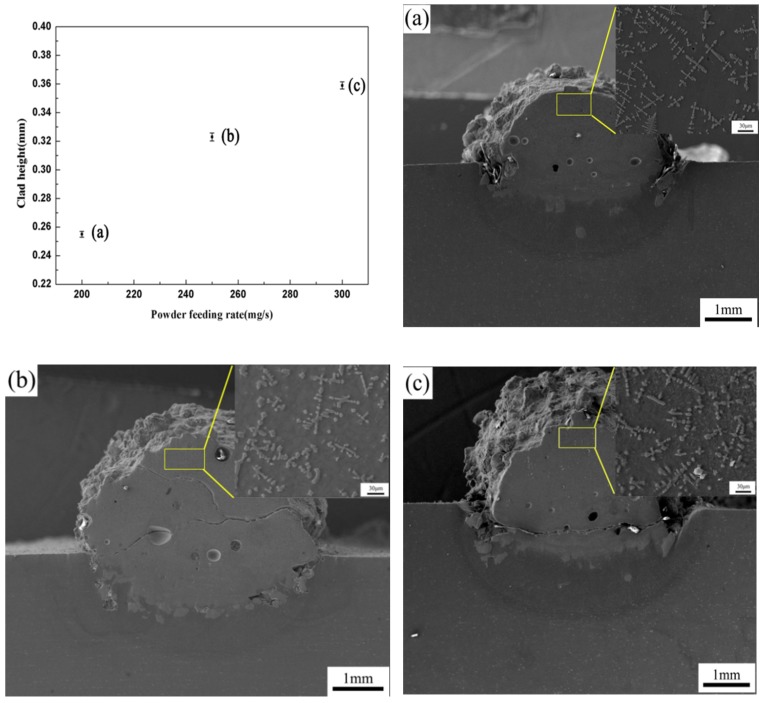
The clad height and microstructure of single clad tracks in different powder feeding rate (F). (**a**) the powder feeding rate of 200 mg/s; (**b**) the powder feeding rate of 250 mg/s; (**c**) the powder feeding rate of 300 mg/s.

Figure 2 gives the correlation between the clad height and P, S and F. The result shows the clad height is controlled by the P^1/4^S^−4/5^F parameter with the linear regression coefficient R^2^ = 0.94 (R = 0.97), which confirms that the statistical linear model is valid. Hence, the main factor influencing the clad height is powder feeding rate. Figure 3 gives the microstructure of single clad tracks in different powder feeding rates when the laser power is 800 W and the scanning rate is 2 mm/s. The result shows the clad height increases with the increasing of the powder feeding rate. It can also be found that the microstructure of coating chiefly comprises of dendrite crystals when powder feeding rate is 200 mg/s (Figure 3a). But the coatings exhibited a cellular dendritic structure when powder feeding rate is 250 mg/s or 300 mg/s (Figure 3b,c). On the one hand, it may be the main reason that TiBCN ceramic powder, which has a high melting point (approximately 3000 °C), cannot melt completely when the laser power is low and the powder feeding rate is high. On the other hand, the solid-liquid interface morphology is dependent on the ratio of temperature gradient to solidification rate (G/R) [27]. It is due to the convectional cooling effects caused by environmental air on the surface of the composite coatings. Thus, the ratio of G/R was small, and a cellular crystal was formed.

**Figure 4:**
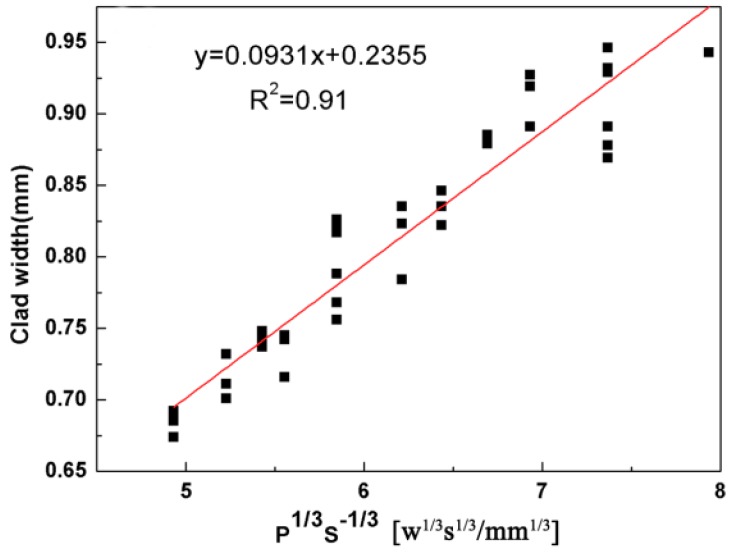
Relationship between the clad width and P^1/3^S^–1/3^.

**Figure 5:**
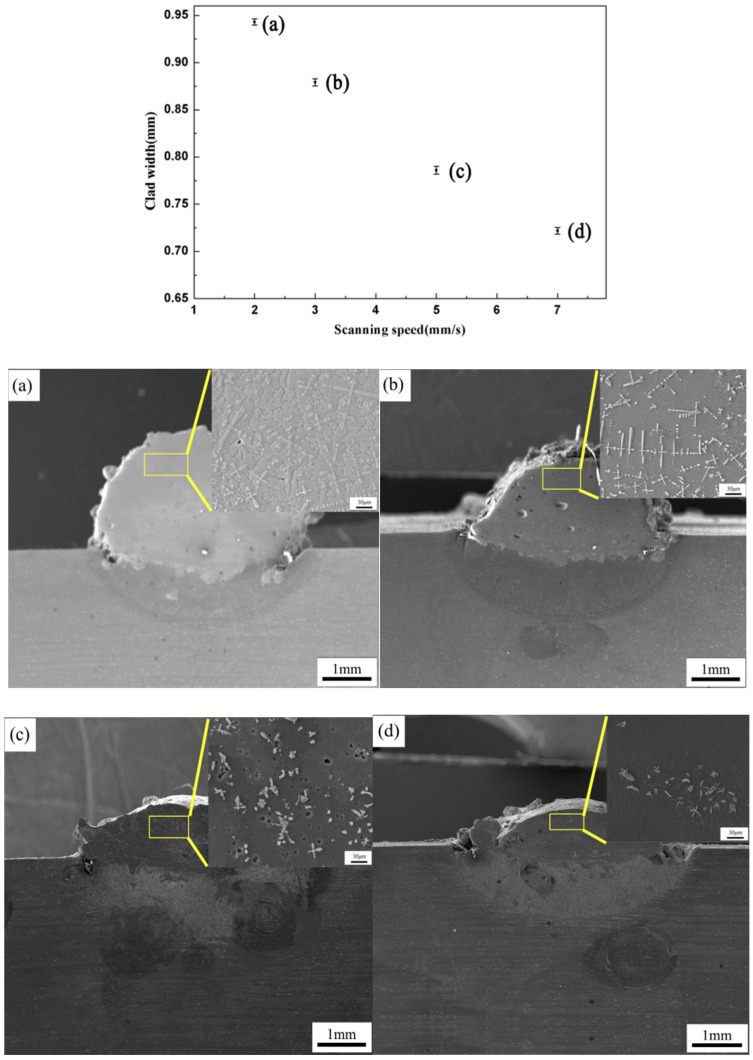
The effect of different scanning speeds on the clad width and microstructure in different scanning speed (S). (**a**) the scanning speed of 2 mm/s; (**b**) the scanning speed of 3 mm/s; (**c**) the scanning speed of 5 mm/s; (**d**) the scanning speed of 7 mm/s.

Figure 4 gives the correlation between the clad width and P, S. It can be concluded that the clad width is controlled by the P^1/3^S^−1/3^ parameter with R^2^ = 0.91 (R = 0.95). The results show that the scanning speed and the laser power are two important parameters. Figure 5 shows the effect of different scanning speeds on the clad width and microstructure when laser power is 1000 W and the powder feeding rate is 300 mg/s. It can be concluded that the clad width decreases when the scanning speed increases. In addition, it can also be seen that the solidification structure changes from a dendritic structure (Figure 5a,b) to a flocculent structure (Figure 5c,d) in the composite coatings. This is attributed to the rapid heating and solidification during laser cladding [28,29]. In other words, the low scanning speed can make the molten pool remain molten over a longer time in higher temperature and remain, resulting in the formation of a dendritic structure.

**Figure 6:**
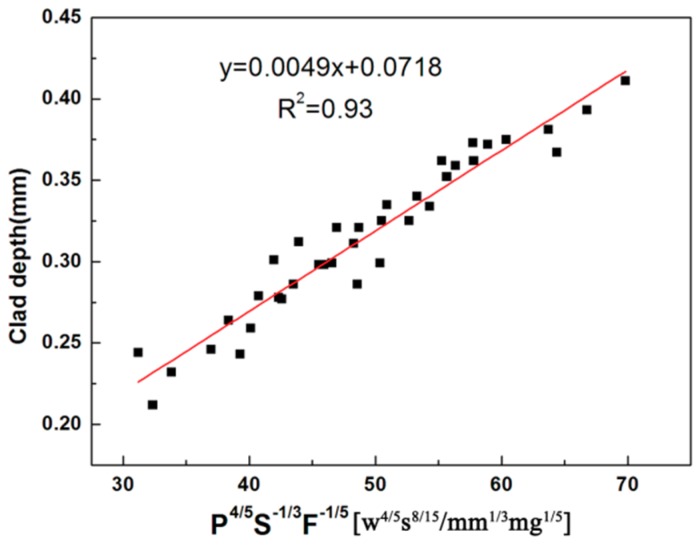
Relationship between the clad depth and P^4/5^S^−1/3^F^−1/5^.

**Figure 7:**
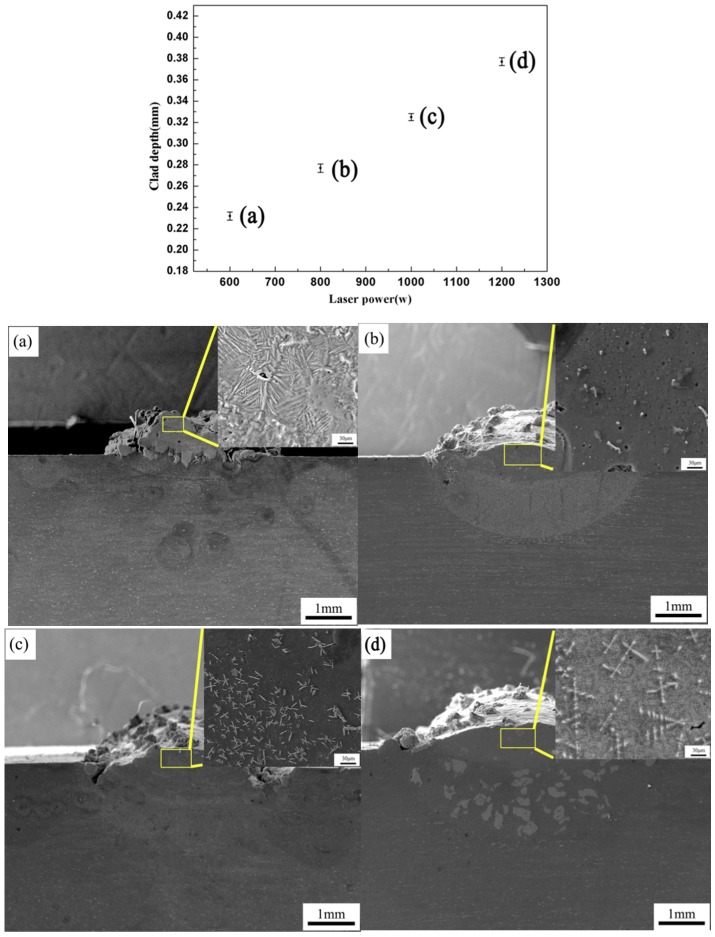
The effect of different laser power on the clad depth and microstructure at different laser power (P). (**a**) The laser power of 600 W; (**b**) the laser power of 800 W; (**c**) the laser power of 1000 W; (**d**) the laser power of 1200 W.

Figure 6 gives the correlation between the clad depth and P, S and F. It can be seen that the clad depth is proportional to the combined parameter P^4/5^S^−1/3^F^−1/5^ with regression coefficient R^2^ = 0.93 (R = 0.96), which confirms that the model is valid. The results show that the laser power is an important parameter. Figure 7 shows the effect of the different laser power on the clad depth and microstructure when scanning speed 5 mm/s and powder feeding rate of 200 mg/s. From Figure 7, it is noted that the clad depth increases when the laser power increases. It can also be found that the unsmoothed surface of the coatings is observed, and has bad metallurgical bonding with the substrate. From Figure 7b, a few particle agglomerations are observed, but not a typical microstructure formation. The microstructure of the coatings changes the flocculent structure Figure 7c to a dendritic structure (Figure 7d) with the increase of laser power.

**Figure 8:**
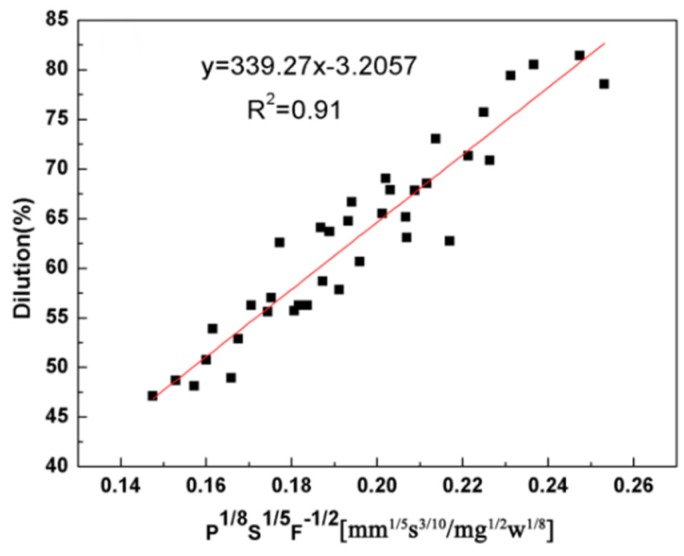
Relationship between dilution and P^1/8^S^1/5^F^−1/2^.

**Figure 9:**
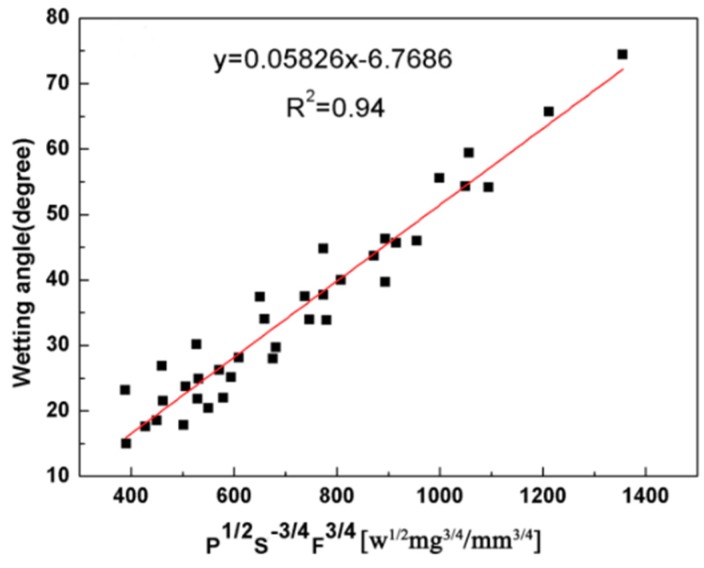
Relationship between dilution and P^1/8^S^1/5^F^−1/2^.

According to reference [26], the dilution rely on the clad height and the clad depth. To avoid the porosity of the cladding coating, the wetting angle is an important parameter. Figure 8 and Figure 9 gives the correlation between the dilution and wetting angle with the three main processing parameters (P, S and F). The dilution is proportional to the combined parameter P^1/8^S^1/5^F^−1/2^ with R^2^ = 0.91 (R = 0.95). From Figure 9, it can be seen that the wetting angle is controlled by the P^1/2^S^−3/4^F^3/4^ parameter with R^2^ = 0.94 (R = 0.96). This indicates laser power and powder feeding rate are indispensable parameters for predicting the dilution and wetting angle, respectively.

**Figure 10:**
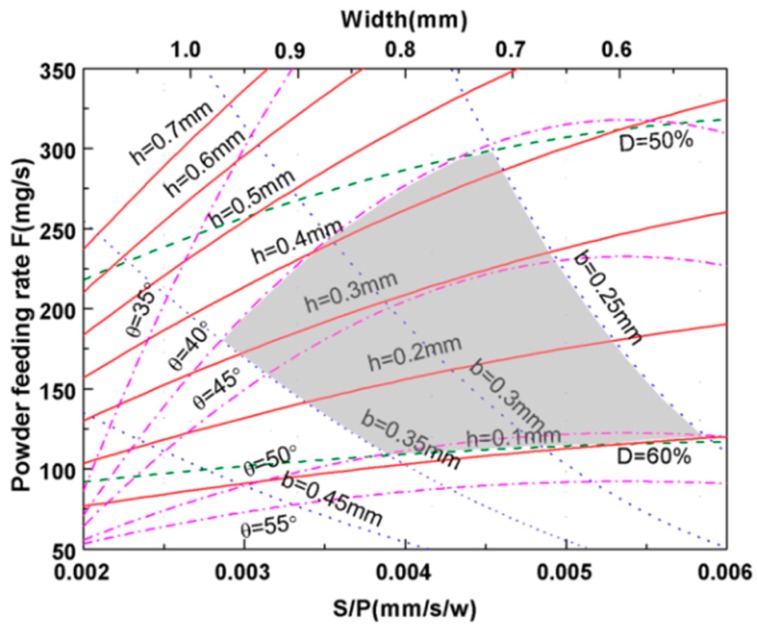
The laser cladding processing map of 85 wt.% Ti + 15 wt.% TiBCN powder on 7075 aluminum alloy substrate in F vs. S/P representation.

Table 3 gives all predicted combined parameters of single clad tracks in the present study. It can be seen from Table 3 that all correlation coefficients show high values (R > 0.95) with their combined parameters. Based on Figure 2, Figure 4, Figure 6, Figure 8 and Figure 9, the laser cladding map of Ti/TiBCN powder on 7075 aluminum alloy substrate is constructed (see Figure 10). The processing parameters zone of forming a good metallurgical bond coating has been marked. According to Figure 10, the optimal processing parameters are as follows: the wetting angle is 40°–50°, the dilution is 50%–60%, the clad height is 0.1 mm–0.4 mm, the clad width is 0.55 mm–0.95 mm, and the penetration depth in the substrate is 0.25 mm–0.35 mm. When Ti/TiBCN powder is fabricated by laser cladding on the 7075 aluminum alloy surface, the process parameters of the shadow area are selected. It acquires a perfect bonded clad coating free of porosity. For instance, it can be predicted that the wetting angle is about 45°, the penetration depth is about 0.3 mm, the dilution is about 55%, and the clad height is approximately 0.3 mm when S/P rate is 0.0037 mm/s/W and powder feeding rate is 250 mg/s. Thus, the optimal parameters are laser power 1000 W, scanning speed 3 mm/s and powder feeding rate 250 mg/s.

### 3.2. Properties

**Figure 11:**
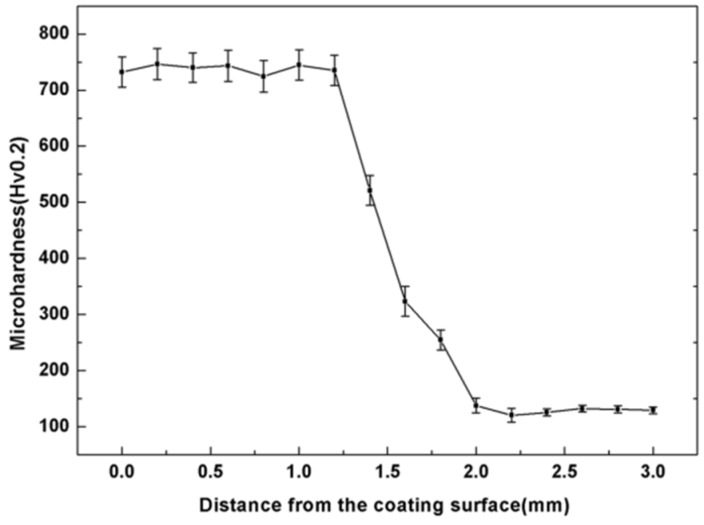
Cross-sectional microhardness profile of Ti/TiBCN composite coating.

**Figure 12:**
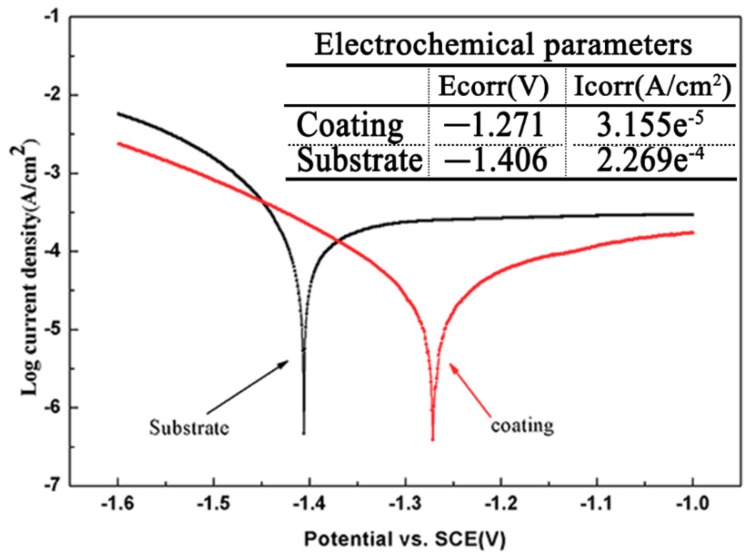
Potentiodynamic polarization curves for the 7075 aluminum substrate and coating.

Figure 11 gives the microhardness change along the depth of the composite coatings. It can be seen that the average hardness value (750 HV_0.2_) was obtained of the Ti/TiBCN composite coating. Figure 12 shows the potentiodynamic polarization curves. It can be seen that the corrosion current density (Icorr) of the Ti/TiBCN composite coating is 3.155 × 10^−5^A/cm^2^. The corrosion potential (Ecorr) for the composite coating is −1.271 V, which is higher than that of the substrate (−1.406 V). Clearly, after cladding with TiBCN powder, the corrosion resistance of the 7075 aluminum has been significantly improved. It indicates that TiBCN powder is a high micro-hardness and corrosion-resistant material.

## 4. Conclusions

The powder feeding rate, the scanning speed and the laser power have a significant effect on the clad height, clad width, clad depth, dilution and wetting angle. These relations could be written in the form P^1/4^S^−4/5^F, P^1/3^S^−1/3^, P^4/5^S^−1/3^F^−1/5^, P^1/8^S^1/5^F^−1/2^ and P^1/2^S^−3/4^F^3/4^ with the correlation coefficients R = 0.97, 0.95, 0.96, 0.95 and 0.96, respectively.

1. The laser cladding processing map of 85 wt.% Ti + 15 wt.% TiBCN powder on 7075 aluminum alloy substrate was designed on the basis of the analysis results. It summarizes an empirical-statistical guideline for selecting processing parameters of laser cladding coating.

2. Compared with the 7075 aluminum substrate, the Ti/TiBCN composite coatings showed higher hardness and corrosion resistance.

## Figures and Tables

**Table 1:** Chemical composition of 7075 Al alloy substrate.

Material	Si	Fe	Cu	Mn	Mg	Cr	Zn	Ti	Al	Others
7075 Al alloy	0.4	0.5	1.2–2.0	0.30	2.1–2.9	0.18–0.28	5.1–6.1	0.2	Bal	<0.05

**Table 2:** The laser parameters used in the present study.

Processing Parameter	Value
Laser power (W)	600–1200
Scanning speed (mm/s)	2–7
Powder feeding rate (mg/s)	200–300
Processing gas Ar shielding gas flow rate (L/min)	2.5
Powder carrier gas flow rate (L/min)	10
Overlap rate	30%

**Table 3:** All predicted combined parameters of single clad tracks in the present study.

Quantity (y)	Combined Paramerer (x)	R	A	B
h (mm)	P^1/4^S^−4/5^F (w^1/4^ mg /mm^4/5^s^1/5^)	0.97	4.29009 × 10^−4^	−0.0181
w (mm)	P^1/3^S^−1/3^ (w^1/3^ s^1/3^ /mm^1/3^)	0.95	0.0931	0.2355
b (mm)	P^4/5^S^−1/3^F^−1/5^ (w^4/5^s^8/15^/mm^1/3^mg^1/5^)	0.96	0.0049	0.0718
D (%)	P^−1/8^S^1/5^F^−1/2^ (mm^1/5^s^3/10^/mg^1/2^w^1/8^)	0.95	339.27	−3.2057
θ (degree)	P^1/2^S^−3/4^F^3/4^ (w^1/2^ mg^3/4^/mm^3/4^)	0.96	0.05826	−6.7686
